# *Vibrio cholerae* can Recycle Fatty Acids Via an Acyl-Acyl Carrier Protein Synthetase

**DOI:** 10.1007/s00284-025-04332-9

**Published:** 2025-06-30

**Authors:** Amanda J. Platt, Amy T. Ma, Joris Beld

**Affiliations:** Department of Microbiology and Immunology, Drexel University College of Medicine, Philadelphia, PA 245 N 15thSt19102 USA

## Abstract

Fatty acids are crucial building blocks for membranes, co-factors, and secondary metabolites, and they are produced by the fatty acid synthase (FAS). Several antibiotics target the bacterial FAS but some bacteria can circumvent FAS inhibition by import and utilization of exogenous fatty acids. The acyl-acyl carrier protein synthetase (AasS) facilitates the direct utilization of fatty acids without the need for breakdown through β-oxidation. Using a combination of unnatural fatty acid supplementation and mass spectrometry we identify here an AasS of *Vibrio cholerae*. In vitro characterization shows that the enzyme can load diverse fatty acids on the FAS acyl carrier protein as well as on coenzyme A. We show that three different FAS-targeted antibiotics can arrest growth of wild type *V. cholerae* and that fatty acid supplementation can rescue this inhibition. In an AasS deletion strain, supplementation with cerulenin and fatty acids allows for growth showcasing the redundancy of environmental fatty acid utilization in *V. cholerae*.

**Supplementary Information:**

The online version contains supplementary material available at 10.1007/s00284-025-04332-9.

## Introduction

Gram negative bacterium *Vibrio cholerae* is the human pathogen responsible for the disease cholera. Although a rare disease in the US, it is still prevalent in developing countries and the WHO reports 3–5 million cases and > 100000 deaths per year [1]. *V. cholerae* is normally found associated with aquatic life (e.g., shellfish) and enters the gastrointestinal tract of humans via contaminated food or water. *V. cholerae* can survive and thrive in many different environments ranging from the ocean to the human gut where the bacteria are exposed to many different molecules, including fatty acids. The concentrations of fatty acids in different environments vary significantly from high concentrations in the mammalian gut (1 kg/L) [2] to low concentrations in ocean water (3 µg/L) [3]. The fatty acid profile also differs between these environments, with polyunsaturated fatty acids in marine environments and both long chain and short chain fatty acids in the human host. Fatty acids can have a range of effects on bacteria. For example, oleic acid (C18:1; Cx:y in which x is the carbon chain length and y the number of unsaturations of the fatty acids) shows anti-virulence activity against some pathogenic bacteria like *Salmonella* [4], while other fatty acids are utilized as nutrient sources. Bacteria can take up fatty acids to be degraded into acetate units and ATP via β-oxidation. In this process, fatty acids first need to be modified into their respective acyl-CoA thioesters before being broken down. Recently it has been shown that some bacteria can efficiently recycle exogenous fatty acids and directly use them to produce new lipids [5].

**Fig 1:**
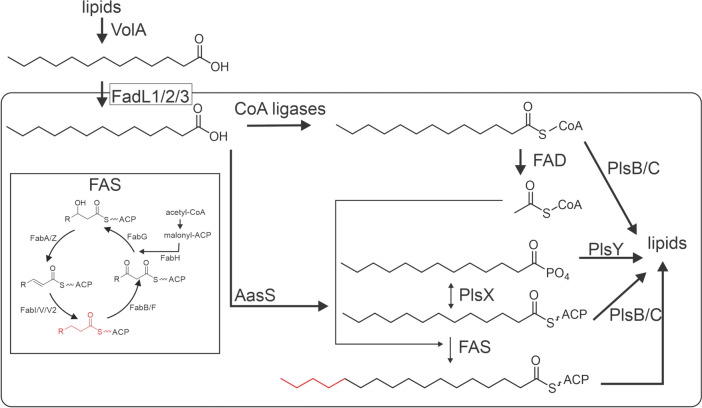
Overview of exogenous fatty acid utilization by *V. cholerae*. Extracellular lipase VolA liberates fatty acids from exogenous lipids and FadL1/2/3 transporters transport fatty acids into the cell. Once in the cell, acyl-CoA ligases and AasSs synthesize acyl-CoAs or acyl-ACPs, respectively. Acyl-CoAs can be degraded via β-oxidation by the fatty acid degradation (FAD) enzymes, or loaded onto lipid headgroups by activity of acyltransferases PlsB/C. Acyl-ACPs are also substrates for PlsB/C. Acyl-ACPs can be further elongated by the fatty acid synthase (FAS, see insert) or transformed into acyl-phosphates by PlsX and subsequently incorporated into lipids by PlsY. The FAS consists of FabH, FabG, FabA/Z, FabI/V/V2 and FabB/F (see Table 1)

Fatty acids are essential for cellular membranes, cofactor and quorum sensing molecule biosynthesis, regulation, and virulence. Fatty acids are biosynthesized de novo by the fatty acid synthase (FAS) but Gram negative bacteria can also take up fatty acids from their environment via FadL transporters in the outer membrane. In contrast to eukaryotes, most bacteria do not accumulate free fatty acids. While eukaryotes encode an acyl-ACP thioesterase, in bacteria fatty acids are not hydrolyzed off the FAS acyl carrier protein (ACP) but directly transferred onto a lipid headgroup. Environmental free fatty acids can have multiple destinations within bacteria; fatty acids can be transformed into acyl-coenzyme As (acyl-CoAs) for lipid biosynthesis or β-oxidation or be loaded directly onto an ACP by an acyl-acyl carrier protein synthetase (AasS) for fatty acid chain elongation by the FAS (Fig. 1). AasSs have only been found and characterized in a few species including *Vibrio harveyi* [6, 7], *Chlamydia trachomatis *[8], *Neisseria gonorrhoeae* [9], *Alistipes finegoldii* [10], cyanobacteria [11] and *Arabidopsis thaliana* [12]. Some Gram-positive bacteria can activate free fatty acids using a kinase and the generated phospho-fatty acids are substrates for lipid biosynthesis acyltransferases PlsX and PlsY (Fig. 1). How bacteria take up and utilize exogenous fatty acids is a topic of very active research, especially with several FAS-targeted antibiotics in the drug-development pipeline [13, 14].

**Table 1:** Ten adenylate-forming enzymes in *V. cholerae.* * Predicted specificity is based on references, Uniprot/Interpro protein annotation (via domain homology) combined with genetic context analysis

Abbrv	Gene	Comments	Predicted specificity*
PrpE	VC1340	No reference but part of typical propionate assimilation cluster	Short chain fatty acids (SCFA)
Acs1	VC0298	RNA-seq on two-component system ‘acetate switch’ CrbRS (VC0303) mutant showed differential regulation of VC0298 [15]	SCFA
Acs2	VCA0829	Unknown. Deletion strain used as a control in QS experiments [16]	SCFA
MenE	VC1971	Present in cluster (operon VC1971-1976) that is upregulated in late infection [17]	O-succinimidyl-benzoic acid
AasS	VC2484	Mentioned in context lipid uptake [18–20]	Long chain fatty acid (LCFA
FadD	VC1985	Necessary for pathogenesis and dissemination in infant rabbit model [21]. Mentioned in context lipid uptake [18, 20]	LCFA
VibE	VC0772	Non-redundant roles of the various iron-scavenging systems [22]; Characterized biochemically as VibE [23]	2,3-dihydroxybenzoic acid
RfbL	VC0249	Putative CoA ligase in the O-antigen surface polysaccharide cluster. RfbL activates and attaches it to either CoA or the carrier protein RfbK. Also known as WbeL [24–26]	2,4-dihydroxybutanoic acid
LcfacL	VC2341	Upregulated in a autoinducer quorum sensing reporter assay [27]	LCFA
DthC	VCA1110	Located in *dth*-region, next to δ-thermostable hemolysin [28]. Mentioned in context lipid uptake [18, 20]	LCFA

Compared to other γ-proteobacteria*, Vibrio* species have many redundant genes both in fatty acid biosynthesis as well as in β-oxidation (fatty acid degradation) (Table S1). It is currently unclear why *Vibrio* species have such redundancy, but it has been shown that *V. cholerae* is a good scavenger of environmental lipids and fatty acids. An extracellular membrane-associated lipase, VolA [18, 29], was found to be responsible for processing extracellular lipids into free fatty acids. Also, when cultures of *V. cholerae* are supplemented with long chain unsaturated fatty acids, they are incorporated into cellular lipids [30, 31]. However, how *V. cholerae* can incorporate exogenous fatty acids into cellular lipids is unknown. We hypothesized that *V. cholerae* encodes for an AasS that confers the ability to directly utilize exogenous fatty acids.

The ability of bacteria to take up exogenous fatty acids might allow them to circumvent FAS-targeted antibiotics. Since the human and bacterial FAS show significant differences, the bacterial FAS has been an attractive target for antibiotics [32]. Cerulenin is a potent inhibitor of ketoacyl synthases, effectively shutting down de novo fatty acid biosynthesis in many bacteria, but it is not an effective inhibitor of the human FAS. Comparing minimal inhibitory concentrations (MICs) for FAS-targeted inhibitors between different bacterial species shows large variations in susceptibility (Table S2), and it has been shown that exogenous fatty acids can rescue FAS inhibition [33]. Growth of *Staphylococcus aureus* inhibited by FAS-targeted triclosan can be rescued by a cocktail of long chain fatty acids [14, 34]. Similarly, when *Streptococcus pneumoniae* was grown in the presence of cerulenin, no growth was observed, but supplementation with long-chain fatty acids at the same time, rescued this phenotype [35]. We showed recently that in *V. cholerae* inhibition of fatty acid biosynthesis by cerulenin can be rescued by addition of a mixture of fatty acids [33]. Here, we interrogated a candidate enzyme involved in this rescue and characterize this AasS using bioinformatic, biochemical and microbiological approaches.

## Results

### Bioinformatic Characterization of Fatty Acid Recycling Genes and Proteins

**Fig 2:**
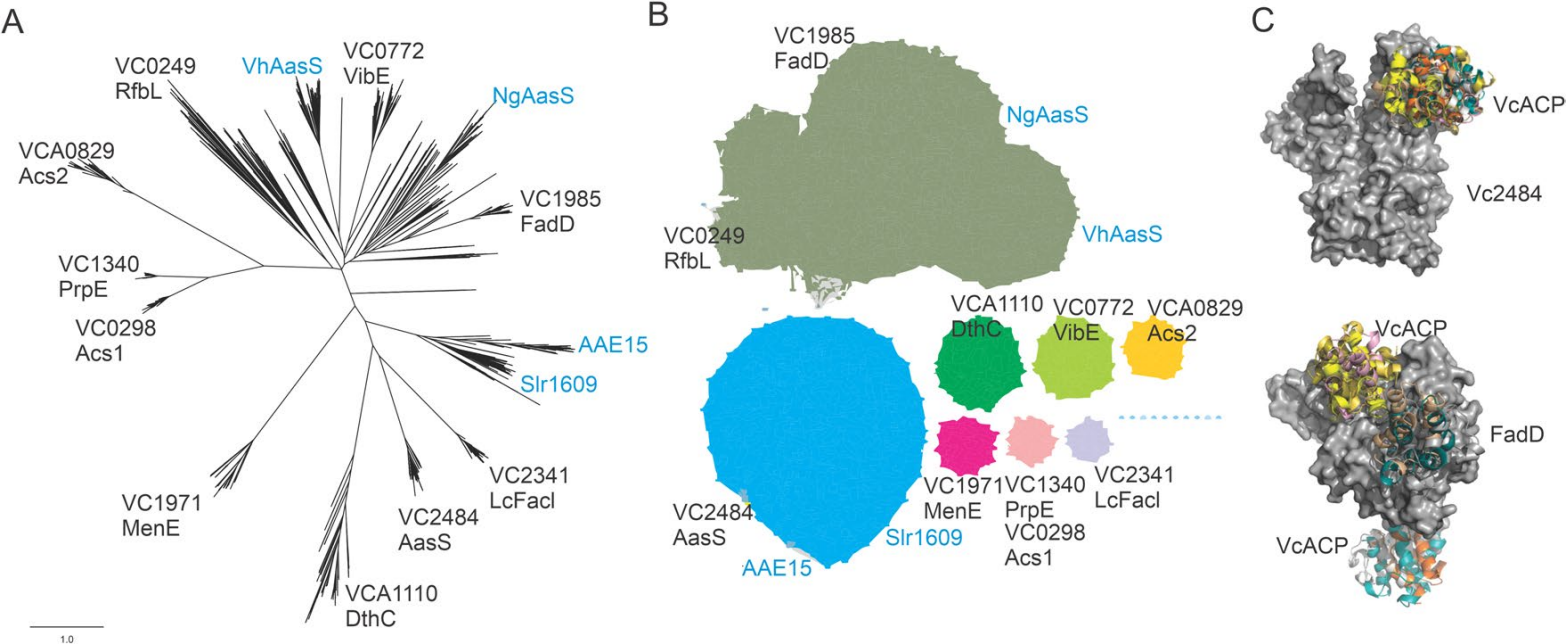
Bioinformatic analysis of adenylate-forming enzymes in *V. cholerae*. **a** Phylogenetic tree and **b** protein similarity network of adenylate-forming enzymes of *V. cholerae* seeded with known AasS enzymes. **c** Docking of VcACP to Vc2484 (top, in gray surface) and Vc1985/FadD (bottom, in gray surface) showing the top 10 models of carrier protein docking to the adenylate-forming enzymes

We used PSI-BLAST of the characterized *V. harveyi* AasS and *Synechocystis* AasS Slr1609 against the *V. cholerae* O1 biovar El Tor str. N16961 genome, to identify candidate AasS enzymes. Ten candidates were found that have CoA ligase or AasS character (Table 1). Systematically we searched these genes in the Uniprot protein database for characterized enzymes with (putative) function. From the candidates only a few have been characterized previously and some are cursorily mentioned in publications (Table 1). Based on sequence alignment alone it is very difficult to identify AasS candidates thus we attempted to bin these enzymes using phylogeny [19] or sequence similarity networks. We constructed phylogenetic trees using MEGA, MUSCLE, and FastTree (Fig. 2a). In the tree VC0249 grouped well with *V. harveyi* AasS, but VC0249 is annotated and characterized as RfbL, a CoA ligase in an O-antigen surface polysaccharide cluster. RfbL activates 2,4-dihydroxybutanoic acid and putatively attaches it to CoA or the carrier protein RfbK [24, 36]. Other candidates included the putative succinyl benzoate CoA ligase VC1971, acyl-CoA ligase FadD involved in β-oxidation, an adenylation domain of the vibriobactin non-ribosomal peptide synthase (VibE), acetyl CoA ligases putatively involved in acetate metabolism, and three other putative CoA ligases. Next, we constructed a sequence similarity network using EFI-EST (Fig. 2b) and observed grouping of different adenylate-forming enzymes in clusters. None of the proteins clustered with VhAasS, but VC2484 showed similarity to AasSs from *Synechococcus* (Slr1609) and *Arabidopsis* (AAE15) (Fig. 2b).

While VC2484 has not been described in the literature, we also came across this gene/protein when analyzing fatty acid metabolism master regulators. Fatty acid biosynthesis and degradation are controlled by several factors including master regulators FabR and FadR, acyl-ACPs and acyl-CoAs [37]. Long chain fatty acid (LCFA) acyl-CoAs and -ACPs bind to FabR and LCFA-CoAs bind to FadR and influence their activity [38]. We analyzed both FadR and FabR regulons using RegPredict [39] and found that FadR is predicted to down-regulate fatty acid transporter FadL1, β-oxidation enzymes FadH/E2/B/A, thioesterase VC2105 (which was discovered by analysis of the FadR regulon [40]), and lipid biosynthesis enzyme PlsB, while activating unsaturated fatty acid biosynthesis enzyme FabA. FabR is predicted to regulate FadL1 and FabA as well as VC2484. In comparison with other bacteria, the latter is a unique putative member of the FabR regulon.

Acyl-CoA ligases act on the small molecule CoA, whereas AasSs act on carrier proteins. Thus, AasSs need to have a protein–protein interaction for successful catalysis whereas acyl-CoA ligases need to bind small molecules. A universal recognition motif or surface patch for carrier protein recognition by partner proteins is not known, but protein structures reveal much about these protein–protein interactions [41].We hypothesized that by computational docking we could visualize differences between the interactions of ACP and these candidate AasSs. When we previously performed docking of ACPs onto dehydratases (e.g., FabA) or ketoacyl synthases (e.g., FabB and FabF), the docked structures and the final structures solved by X-ray crystallography overlap well [42]. We constructed homology models of both *V. cholerae* ACP, and putative AasSs using the Swissmodel server. Docking using Cluspro of VcACP onto models of VC2484 showed a tight protein–protein interface between VC2484 and ACP. In contrast, docking of ACP to VC1985, which encodes the LCFA acyl-CoA ligase FadD, showed random binding of ACP onto the protein (Fig. 2c).

### Fatty Acid Recycling by acyl-CoA Ligase/AasS Transposon Mutants

**Fig 3:**
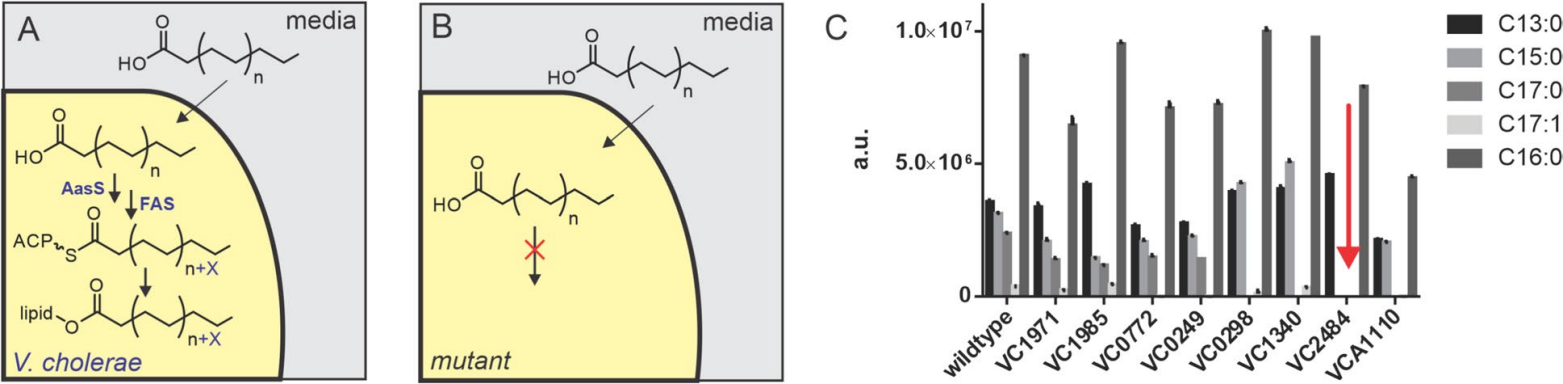
Fatty acid supplementation and GCMS analysis of *V. cholerae* transposon mutants. **a** Wildtype *V. cholerae* can take up odd-chain fatty acids, load them onto ACP via an AasS, and directly use them for fatty acid biosynthesis. **b** a transposon mutant in AasS cannot load exogenously supplied fatty acids onto ACP. **c** Fatty acid methyl ester analysis by GCMS of several transposon mutants. Tridecanoic acid (C13:0) was supplemented to *V. cholerae* and extended fatty acids observed via GCMS. Palmitic acid (C16:0) is included as a reference

Although these bioinformatics experiments reveal much about the presence of these adenylate-forming enzymes in *V. cholerae*, in vitro experiments are required to unambiguously show enzymatic activity. We used mutants from a transposon mutant library to screen for putative AasS activity [43]. We supplemented different mutant strains with tridecanoic acid (C13:0) and analyzed fatty acid profiles of bacteria grown in the presence of this fatty acid by gas chromatography mass spectrometry (GCMS). Odd-chain fatty acid supplementation is useful since odd-chains are rare in nature, and thus facile to detect by GCMS [44]. By screening AasS candidates in the transposon mutant library we hypothesized that an AasS mutant would not be able to elongate the fed fatty acids, and thus we would not observe C15, C17, or C19 acids (Fig. 3). Wildtype *V. cholerae* produces very small amount of C13:0, C15:0, C17:0 (< 1% [45]) under standard growth conditions, whereas a culture supplemented with C13:0 shows significant amounts of these acids, demonstrating that *V. cholerae* can take up and extend exogenous fatty acids (Fig. 3). Some of the mutants (*acs1*, *prpE*, and *vca1110*) show formation of C15:0 but not C17:0, which could be due to altered regulation of acyl chain extension length, or differences in fatty acid profile homeostasis. The *vc2484* mutant shows no formation of C15:0 or C17:0, suggesting that VC2484 is an AasS.

### In Vitro Characterization of VC2484

**Fig 4:**
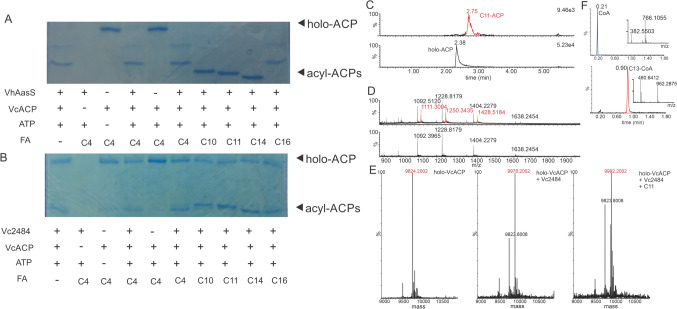
VC2484 loads VcACP and coenzyme A with fatty acids. VcACP was incubated with either **a** VhAasS or **b** VC2484. Using 20% Urea-PAGE holo- and acyl-ACPs can be resolved based on the different shape of the carrier protein. Whereas VhAasS efficiently loads fatty acids onto VcACP, VC2484 is less efficient in loading the fatty acids onto VcACP. Both AasSs load fatty acid contaminants onto VcACP. **c** VcACP was incubated with VC2484 and C11 and after o/n incubation at 37 °C a peak for holo-VcACP (at 2.38 min) and C11 loaded VcACP (at 2.75 min) can be observed, in a relative ratio of 5:1, in accordance with the gel shift activity assay. **d** Mass spectra of a mixture of holo- (black) and C11-loaded (red) VcACP. **e** Deconvolution of the ESI mass spectra and comparison of the masses show a mass of 9824 Da for holo-VcACP, 9978 for loading of a contaminating fatty acid and 9992 Da for loading of C11. The difference in mass between C11 loaded VcACP and holo-VcACP is 168 Da, which corresponds to the C11 acyl group attached to the phosphopantetheine arm. Calculating the difference for the control reaction in the absence of added fatty acids, shows a difference of 154 Da, which corresponds to decanoic acid. **f** CoA ligase activity of VC2484 determined by incubating VC2484 with CoA, tridecanoic acid (C13) and ATP (bottom). In the absence of VC2484 (top) no CoA ligation is observed

We cloned the *acpP* and *vc2484* genes from *V. cholerae* genomic DNA into *E. coli* expression vectors (pET29a) and transformed the plasmids into *E. coli* BL21 [DE3]. Protein expression and purification via hexahistidine affinity tag purification gave us VC2484 and VcACP as shown by SDS-PAGE and intact protein mass spectrometry. We used conformationally sensitive Urea-PAGE [46] to determine the ratio of apo- and holo-VcACP. Urea-PAGE is a semi-native, semi-denaturing gel electrophoresis technique in which apo/holo/acyl-ACPs can be separated. Since the fatty acid is buried in the inner-core of the ACP, acylated-ACPs are more compact and run faster through the acrylamide gel. The endogenous 4’-phosphopantetheinyl transferase (PPTase) of *E. coli* (AcpS) is known to post-translationally modify FAS carrier proteins from a variety of organisms and indeed we observe a mixture of apo- and holo-VcACP, when this ACP is expressed in *E. coli.* We assayed the ability of VhAasS and VC2484 to catalyze acylation of holo-VcACP using UREA PAGE. VhAasS efficiently acylates VcACP with various fatty acids, except C16 (Fig. 4a). Incubating VC2484 with VcACP and C10, C11, C14, and C16 resulted in the appearance of new bands traveling further in the gel, suggesting more compact species (Fig. 4b). By LCMS a new peak with the mass of VcACP loaded with C11 can be seen (Fig. 4c–e). VC2484 is able to load different chain length fatty acids onto VcACP, albeit not as efficiently as VhAasS (Fig. 4b vs 4a). As we observed previously, both AasSs load contaminating fatty acids onto ACP even without the addition of fatty acids (lane 1 of Fig. 4a and lane 1 of Fig. 4b). While VhAasS seems to load contaminating short chain fatty acids, VC2484 loads a medium or long chain fatty acid. When we analyze the reaction of holo-VcACP with VC2484 and C11, we detect holo-VcACP and C11 loaded ACP (Fig. 4c–e), whereas a reaction without added fatty acid, shows a signal for C10-modified ACP. Using LCMS, we have also shown that VC2484 is able to load fatty acids onto coenzyme A itself (Fig. 4f). While some characterized AasS enzymes show acyl-CoA synthetase activity [47], others show none-to-little [48]. Thus far, no acyl-CoA synthetases have been characterized that also have AasS activity.

### Decanoic Acid is Toxic to *V. cholerae*

Different fatty acids can have various effects on bacteria, ranging from toxicity to being used as nutrients. We systematically supplemented cultures of wildtype, a *vc2484* transposon mutant, and a complemented strain with various concentrations of different fatty acids (Fig. S1). As previously observed, C10 is toxic to *V. cholerae* [49]. Interestingly in this assay, C10 and C11 were more toxic to the *vc2484* transposon mutant than the wild type, while C3, C16:0, and C18:1 show no growth phenotype difference.

### Effects of Fatty Acid Synthase Inhibitors

**Fig 5:**
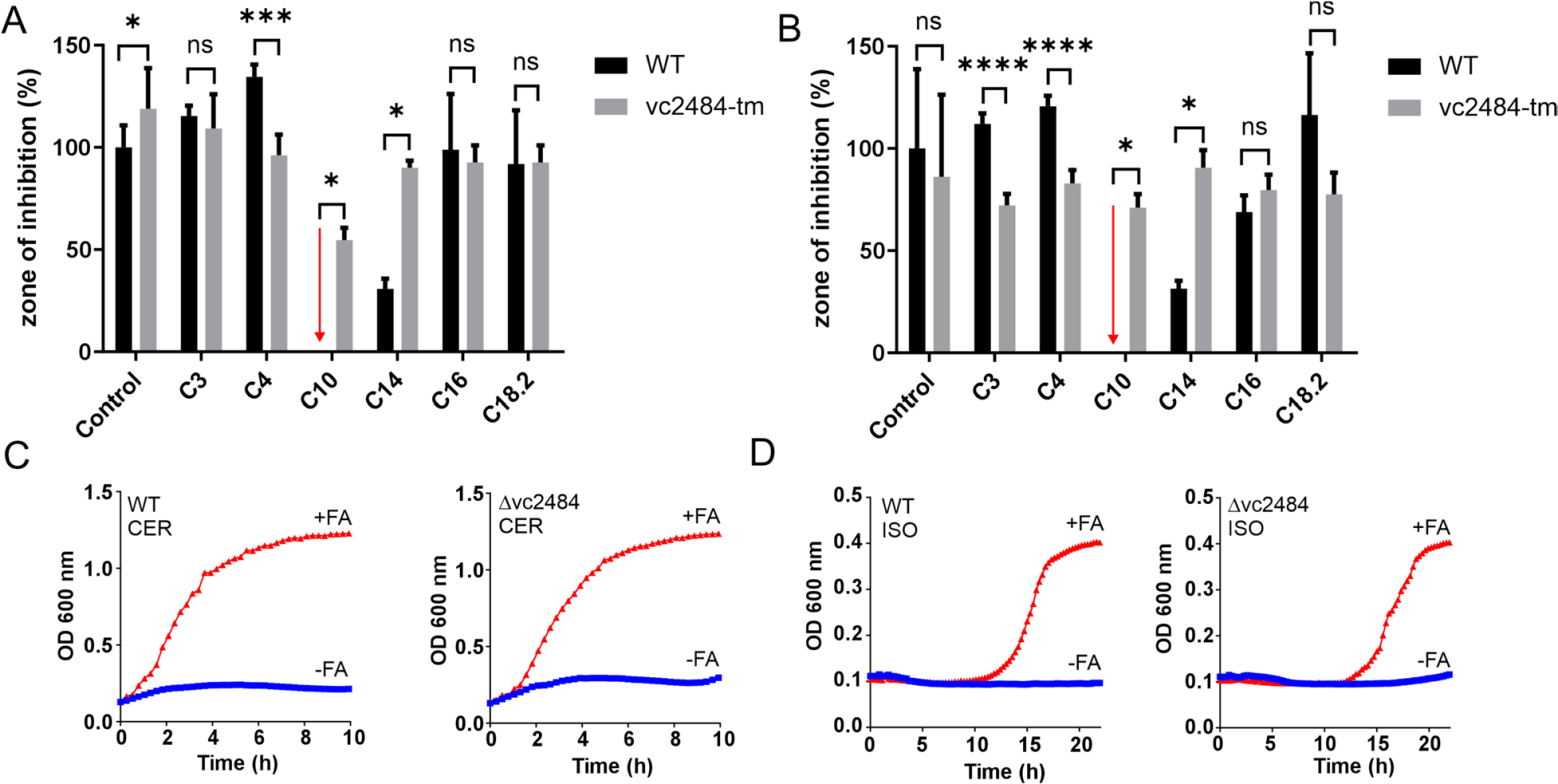
Rescue of FAS inhibition by fatty acids. **a** Isoniazid and **b** triclosan inhibitors were spotted on a sterile filter disk on a lawn of WT or *vc2484* mutant bacteria growing on M9 media plates supplemented with 1 mM fatty acids. If fatty acids can rescue the FAS inhibition, a smaller halo surrounding the spotted inhibitor would be observed. Decanoic acid (C10) prohibits all growth of wild type (WT) bacteria (arrow), but the *vc2484* transposon mutant shows growth in the presence of this fatty acid. Myristic acid (C14) rescues growth of wild type and a smaller halo is observed. Other fatty acids do not seem to rescue FAS inhibition in this assay. (Unpaired *t*-test in Graphpad Prism 8 was used for statistics, **** *p* < 0.0001, *** *p* < 0.001, * *p* < 0.05) **c** Growth inhibition by 5 µM cerulenin (in LB media) or **d** 10 mM isoniazid (in M9 media) is rescued in WT and *vc2484* mutant by addition of a mixture of fatty acids (1 mM)

Next, we set out to interrogate the ability of *V. cholerae* to circumvent FAS-targeted inhibitors by utilizing exogenous fatty acids via AasS. Although several FAS enzymes have been targeted with anti-bacterials, relatively little is known about FAS-targeting in *V. cholerae*. Cerulenin is an inhibitor of FAS ketoacyl synthases and is efficient in inhibiting bacterial fatty acid biosynthesis [50]. Reported MICs for cerulenin in different bacteria range between 1.5–100 µg/ml (see Table S2). Cerulenin was used in stringent response assays to starve *V. cholerae* of fatty acids at 200 µg/ml [51]. We show here concentration-dependent inhibition by cerulenin (Fig. S2). Next, we supplemented *V. cholerae* with FAS inhibitors, fatty acids and mixtures of fatty acids. Whereas growth of *V. cholerae* is inhibited by cerulenin, additional supplementation with a mixture of fatty acids rescues growth (Fig. 5c, d and [33]).

Although isoniazid has been used extensively to eliminate *Mycobacterium tuberculosis* by inhibiting the enoyl-ACP reductase InhA of the fatty acid synthase (with an MIC of 0.2 µg/ml), very few studies have discussed the ability of this molecule to inhibit other enoyl reductases. When tested against *E. coli* or *Salmonella typhimurium,* MIC values are reportedly > 500 µg/ml, while isoniazid inhibits growth of *V. cholerae* with a MIC > 150 µg/ml (Fig. S2). We wondered whether isoniazid targets one or several of the three enoyl reductase isoenzymes (FabV1, FabV2 or FabS) present in *V. cholerae*. It has been established that FabV1 is essential, whereas FabV2 and FabS are not [52]. We tested FabS and FabV2 transposon mutants in an inhibition assay with isoniazid and observed no difference with wild type (Fig. S3). Having established isoniazid as a FAS inhibitor in *V. cholerae*, we next determined whether VC2484 is crucial for environmental fatty acid scavenging when endogenous fatty acid biosynthesis is inhibited.

We attempted to rescue growth inhibition by isoniazid and triclosan spotted on sterile disks on lawns of bacteria on agar plates that were supplemented with different fatty acids (Fig. 5). In this assay, decanoic acid (C10) is toxic to wild type but less so to a *vc2484* transposon mutant. Myristic acid (C14) seems to be able to rescue growth of isoniazid or triclosan treated wild type but not the *vc2484* transposon mutant. Next, we treated wild type and ∆*vc2484* with a combination of an inhibitory concentration of isoniazid or cerulenin and a mixture of fatty acids in liquid culture, and both wild type and ∆*vc2484* could be rescued by fatty acid supplementation (Fig. 5c, d), suggesting redundancy in exogenous fatty acid utilization.

## Discussion

Fatty acid biosynthesis is an essential pathway for nearly all organisms, forming the building blocks for membranes and several co-factors [42]. Although the enzymatically catalyzed chemical reactions of both human and bacterial fatty acid synthases are identical, the proteins and enzymes involved are substantially different, and thus the bacterial FAS can be targeted with antibiotics [53]. Some bacteria however can obviate the need for de novo biosynthesis by scavenging fatty acids from their environment [54, 55]. One route for direct utilization of exogenous fatty acids is via the activity of the adenylate-forming enzyme AasS. *V. cholerae* encodes for ten adenylate-forming enzymes that activate various substrates including acetate, propionate, and long chain fatty acids, for loading onto CoA or a carrier protein. We used a combination of bioinformatic and in vitro approaches to identify an AasS that can assist in the utilization of exogenously supplied fatty acids, and that in vitro can load fatty acids onto the FAS acyl carrier protein. The in vitro activity of the *V. cholerae* enzyme for the loading of various chain lengths of fatty acids onto ACP is relatively poor in comparison to the AasS from *V. harveyi* (Fig. 4). But previous work on plant, bacterial, and cyanobacterial AasSs has shown large differences in the in vitro activity of these enzymes. Perhaps this is a reflection of the in vivo activity, or in vitro conditions are a poor mimic of the intracellular conditions and poor in vitro activity is for example due to instability of the protein, or product inhibition.

The presence of exogenous fatty acid recycling mechanisms might allow bacteria to escape inhibition of their de novo fatty acid biosynthesis which could potentially hinder the effectiveness of FAS targeting antibiotic compounds [56]. This has been studied best in Gram-positive *S. aureus* that encodes for fatty acid kinases [5, 9, 53]. Exogenous fatty acids can rescue triclosan treatment of *S. aureus* in a fatty acid kinase dependent manner [14, 34]. Growth of the Gram negative bacterium *Neisseria gonorrhoeae* is inhibited by targeting the FAS with AFN-1252. Although *N. gonorrhoeae* encodes an AasS [9], supplementation with a combination of C16/C16:1 did not rescue growth, suggesting that either exogenous fatty acids cannot rescue FAS inhibition or that the mixture of these two C16 fatty acids cannot rescue FAS inhibition. Since several metabolic and regulatory processes depend on fatty acids it might be that a mixture of C16/C16:1 cannot substitute for the fatty acid requirements of the bacteria.

Here, we show that commonly used FAS inhibitors also inhibit the growth of *V. cholerae* albeit at MICs higher than for other bacteria (Table S2). We speculate that the redundancy of fatty acid biosynthesis [57], uptake, and degradation proteins influence the effectiveness of FAS inhibitors. We also show that a mixture of fatty acids can rescue growth of *V. cholerae* treated with a high concentration of FAS inhibitor (Fig. 5c, d). Previously, it has been shown that in *Chlamydia*, *Neisseria*, and *Staphylococcus* species FAS inhibition cannot be rescued by supplementation with fatty acids, whereas *Streptococcus agalactiae* can grow with deletions in the endogenous fatty acid synthesis genes when supplemented with fatty acids [5]. When we used a *vc2484* deletion strain we could not show dependency of this rescue on the presence of this gene (Fig. 5c, d).

We speculate that *V. cholerae* has redundancy in its exogenous fatty acid utilization mechanisms, allowing a *vc2484* mutant to grow in the presence of cerulenin and fatty acids. *V. cholerae* encodes for at least 10 different adenylate-forming enzymes. While a *vc2484* transposon mutant showed complete disappearance of C15 and C17 when cultured in the presence of C13, suggesting that C13 cannot be loaded onto the ACP and participate in FAS-catalyzed chain extension, also transposon mutants in *acs1*, *prpE*, and *vca1110* were affected (Fig. 3). Although their prime activity is perhaps not activation of taken-up fatty acids, substrate promiscuity might allow them to activate these fatty acids as acyl-ACPs or -CoAs. Alternatively, while we focus here on AasS enzymes, taken-up fatty acids can also be transformed into acyl-CoAs, which can be used by PlsB/C for lipid biosynthesis. Thus, deletion of just one AasS is perhaps not sufficient to eliminate exogenous fatty acid utilization. Interestingly, our AasS enzyme inhibitor is active in combination with cerulenin when wild type *V. cholerae* is grown in the presence of fatty acids [33], suggesting that this inhibitor targets multiple adenylate-forming enzymes. In fatty acid metabolism, it is common to have functional redundancy, likely for fine-tuning of fatty acid profiles [57], or to evade antibiotic treatment. For example, *V. cholerae* encodes for four enoyl-ACP reductases (FabI, FabV, FabV2 and FabS) from which only FabI is sensitive to triclosan [58]. Here, likely multiple proteins can assist in utilizing exogenous fatty acids.

When we tested toxicity of fatty acids on wild type and the deletion strain, we found that decanoic acid is toxic to *V. cholerae*. While in liquid culture C10 seems more toxic to a *vc2484* transposon mutant than wild type, on solid media C10 could rescue growth of a FAS-inhibited wild type, but not that of a *vc2484* transposon mutant (Fig. 5). We hypothesize that there is redundancy in the exogenous fatty acid utilization mechanism in *V. cholerae*. In wild type cells, uptake of C10 leads to loading of C10 onto the acyl carrier protein, shifting the pool of acylated-ACPs. This shift influences cellular processes that are dependent on acylated-ACPs, like fatty acid, lipid, lipoic acid and biotin biosynthesis. The unique toxicity of decanoic acid has been observed by others but its mechanism remains unknown [49, 59].

*V. cholerae* has an intimate relationship with fatty acids in its environment. For example, recently it was shown that cholera toxin induces long chain fatty acid release from the host, which the bacteria utilize for proliferation [60, 61]. Subsequently, long chain fatty acids also bind to master regulator ToxT, repressing the production of virulence factors. In this way, the bacterium senses its environment and tunes its pathogenicity. Here we present a mechanism for fatty acid utilization that supports combined targeting of fatty acid biosynthesis and fatty acid recycling enzymes as an attractive antibiotic approach [33, 47, 62, 63].

## Experimental Procedures

### Bioinformatics

*Prediction regulons*. FabR and FadR regulons were predicted using RegPredict. *Phylogenetic trees*. Sequences for *V. harveyi* AasS, *N. gonorrhoeae* AasS, Slr1609 and the ten adenylate-forming enzymes of *V. cholerae* were downloaded from NCBI and each PSI-BLASTed against the nr database of the NCBI. The top 100 hits for each enzyme were combined and aligned using MUSCLE and a phylogenetic tree was built using FastTree or MEGA. Trees were visualized using Figtree and Dendroscope. *Protein similarity networks*. Networks were constructed from the same dataset using EFI-EST and visualized using Cytoscape. *Protein–protein docking*. Protein structure homology models were constructed using Swissmodel and protein–protein docking was conducted on the Cluspro server. We also used Alphafold 2 for protein and complex modeling and observed similar results (data not shown). Structures were visualized using Pymol.

### Cloning

*Deletion of vc2484*. The deletion mutant in *V. cholerae* strain C6706 was constructed via well-established allelic exchange methods with the pWM91 suicide vector [64] containing an R6K origin of replication [65]. Briefly, flanking PCR products were assembled into pWM91 using isothermal assembly cloning techniques, transformed into *E. coli* DH5α λpir, and verified by restriction digest. After transformation into SM10 λpir, the donor strain was conjugated with *V. cholerae* and exconjugants were isolated on LB agar plates containing streptomycin and ampicillin, each at 100 μg/mL. After counterselection with sucrose, ampicillin sensitive colonies were screened for their respective gene deletions by PCR. A complementation vector (in arabinose inducible pBAD33) was constructed via isothermal assembly and conjugated into *V. cholerae *∆*vc2484*. *Expression constructs*. Expression constructs for VcACP, VhAasS and VC2484 were constructed using isothermal assembly in pET16b and pET29a.

### GCMS and LCMS

*Gas chromatography mass spectrometry (GCMS)*. Bacterial pellets of *V. cholerae* WT and transposon mutants grown (o/n at 37 °C with continuous shaking) in 5 ml of M9 media supplemented with glucose (0.2% w/v) and tridecanoic acid (100 µM) were washed with M9 media, treated with 1 M HCl in methanol for 1 h at 65 °C and the fatty acid methyl esters (FAMEs) extracted using hexanes. Samples were analyzed on an Agilent 7890B/5877A system equipped with a 122–2362 DB 23 (60 m × 250 um × 0.25 um) column. Samples (1 µl) were injected in splitless mode and the inlet was kept at 220 °C. Initial oven conditions were 60 °C and the temperature ramped at 45 °C/min to 200 °C, 20 min at 200 °C, followed by 20 °C/min to 220 °C and kept at 20 min. Agilent Masshunter was used to extract FAME data from the raw data.

### Liquid Chromatography Mass Spectrometry (LCMS)

Proteins and acyl-CoAs were analyzed on a Waters Acquity I-Class UPLC system coupled to an Acquity TUV detector and Synapt G2Si HDMS mass spectrometer in negative ion mode with a heated electrospray ionization (ESI) source in a Z-spray configuration. LC separation was performed on a Waters Acquity UPLC BEH 1.7 µm 2.1 × 50 mm column using an 0.5 ml/min gradient of 99/1 to 5/95 in 10 min followed by washing and reconditioning the column. Eluent A is 10 mM ammonium formate (pH 4.4) in water/acetonitrile and B is 10 mM ammonium formate (pH 4.4) in acetonitrile/isopropanol. Conditions on the mass spectrometer were as follows: Capillary voltage 2 kV, sampling cone 30 V, source offset 80 V, source 150 °C, desolvation 400 °C, cone gas 0 L/h, desolvation gas 900 L/h and nebulizer 6.5 bar. MS^e^ data were collected using a ramp trap collision energy 20–40 V, and masses were extracted from the TOF MS TICs using an abs width of 0.005 Da.

### Fatty Acid Supplementation and FAS Inhibitor Experiments

Bacterial growth curves were collected on a Tecan M200Pro platereader in 96-well plates shaking at 37 °C and absorbance measured at 600 nm. A total of 200 µl of LB or M9 medium with and without supplements were aliquoted per well. Overnight *V. cholerae* cultures grown in LB were inoculated at a 1:1000 dilution. The mixture of fatty acids contained short chain and long chain fatty acids (C3, C4, C14, C16, C18, C18:1). All experiments were performed in biological duplicate and repeated twice. For fatty acid toxicity tests, we spotted inhibitors on a sterile filter disk on a lawn of WT or *vc2484* mutant on M9 media plates supplemented with 1 mM fatty acids.

### Protein Expression and Purification

*E. coli* BL21[DE3] harboring pET29a-VcACP or pET16b-VC2484 were grown for 6 h at 37 °C, prior to 12 h induction with 500 μM IPTG, at room temperature. Cells were harvested by centrifugation, resuspended in lysis buffer (500 mM sodium acetate, 50 mM phosphate buffer, 20% glycerol and 0.1 mM EDTA) and sonicated six times for thirty second intervals at 60% amplitude. Cell debris was spun down for 1 h at 10000 rpm and 4 °C. The crude lysate was applied to a pre-equilibrated Ni–NTA column and incubated for 15 min at room temperature. The resin was washed extensively (20 ml) and the protein eluted with 100 mM imidazole in 20 mM phosphate buffer, containing 20% glycerol, 100 mM sodium acetate and 0.1 mM EDTA. The protein was dialyzed against PBS containing 20% glycerol prior to storage at − 80 °C. Sfp was expressed and purified as previously described. Apo-VcACP was treated with Sfp in the presence of CoA and MgCl_2_ and the mixture used as is.

### In Vitro Activity Assay

Sfp (3 µM), VcACP (3 µM), coenzyme A (3 µM), magnesium chloride (25 mM), dithiothreitol (1 mM), and sodium phosphate buffer pH 8.0 (50 mM) were combined and incubated at 37 °C for 2 h to facilitate loading of the PPant arm. Next, ATP (1 mM), triton-X (0.1%), fatty acid (100 µM), and Vc or Vh AasS were added and mixtures were incubated at 37 °C for an additional 18 h. Ten µl were removed and resolved on a 20% urea PAGE gel. The remaining 10 µl were used for LCMS verification.

## Supplementary Information

Below is the link to the electronic supplementary material.

Supplementary file1 (DOCX 2509 KB)
