# Supplementary material for: Vibrio cholerae can Recycle Fatty Acids Via an Acyl-Acyl Carrier Protein Synthetase
Source: Curr Microbiol. 2025 Jun 30;82(8):352. doi: 10.1007/s00284-025-04332-9 (PMC12208996; doi:10.1007/s00284-025-04332-9)
Supplement: Supplementary file 1 — Supplementary file1 (DOCX 2509 KB) [file 284_2025_4332_MOESM1_ESM.docx]

***Supplemental Information***

***Vibrio cholerae* can recycle fatty acids via an acyl-acyl carrier protein synthetase**

Amanda J. Platt^a^, Amy T. Ma^b^ and Joris Beld^*a^

^a^Department of Microbiology and Immunology, Drexel University College of Medicine, 245 N 15^th^ St, Philadelphia, PA 19102, USA

^b^Department of Microbiology, Perelman School of Medicine, University of Pennsylvania

*Correspondence: jb3669@drexel.edu. Phone: +1 267 359 2355.

Table S1 – Fatty acid and lipid metabolism proteins in *V. cholerae*

Table S2 - Minimal inhibitory concentrations of various FAS-targeted inhibitors.

Figure S1 - Representative growth curves of fatty acid supplementation to V. cholerae wild type, vc2484 transposon mutant, and the same transposon mutant complemented.

Figure S2 – Growth curves of V. cholerae in the presence of FAS inhibitors.

Figure S3 - Growth curves of V. cholerae WT, FabV2, and FabS mutants in the presence of isoniazid.

Table S1

| Table S1 – Fatty acid and lipid metabolism proteins in *V. cholerae* | | | | |
| --- | --- | --- | --- | --- |
| Pathway | Abbrv | Name | Gene | Ref |
| FAS | AcpP / ACP | Acyl carrier protein | VC2020 | [1, 2] |
|  | FabD / MCAT | Malonyl CoA acyltransferase | VC2022 | - |
|  | FabH / KS3 | Ketoacyl ACP synthase (initiation) | VC2023 | [2] |
|  | FabF / KS2 | Ketoacyl ACP synthase (extension) | VC2019 | [2] |
|  | FabB / KS1 | Ketoacyl ACP synthase (extension) | - |  |
|  | FabG / KR | Ketoacyl ACP reductase | VC2021 | [3] |
|  |  |  | VC1591 / AldO | [4-6] |
|  | FabA / DH | Acyl ACP Dehydratase | VC1483 | [2] |
|  | FabZ / DH | Acyl ACP Dehydratase | VC2249 | - |
|  | FabI / ER | Enoyl ACP reductase | - |  |
|  | FabV / ER | Enoyl ACP reductase | VC1738 | [7] |
|  | FabV2 / ER | Enoyl ACP reductase | VCA0784 | [7, 8] |
|  | FabS / ER | Enoyl ACP reductase | VC2093 | [9] |
|  | FabR | Regulator | VC0152 | [10] |
|  |  |  |  |  |
| FAD | FadL1 | Transporter | VCA0862 | [11] |
|  | FadL2 | Transporter | VC1043 | [12] |
|  | FadL3 | Transporter | VC1042 | [13] |
|  | FadD | Acyl-CoA ligase | VC1985 | [12] |
|  | FadE | Acyl-CoA dehydrogenase | VC2231 | [13] |
|  |  |  | VC1740 | [13] |
|  | FadB | Acyl-CoA dehydrogenase | VC2758 | [2, 14] |
|  | FadA | Acyl-CoA thiolase | VC2759 | [14] |
|  |  |  | VCA0690 | [15] |
|  | FadJ | Acyl-CoA dehydrogenase | VC1047 | [14] |
|  | FadI | Acyl-CoA thiolase | VC1046 | [14] |
|  | FadR | Regulator | VC1900 | [16] |
|  | VC2105 | Thioesterase | VC2105 | [17] |
|  |  |  |  |  |
| Lipid | VolA | Extracellular lipase | VCA0863 | [11] |
|  | PlsB | Acyltransferase | VC0093 | [[49](#_ENREF_49) |
|  | PlsC | Acyltransferase | VC2513 | - |
|  | PlsX | Acyltransferase | VC2024 | - |
|  | PlsY | Acyltransferase | VC0523 | [3] |
|  |  |  |  |  |

Table S2

| Table S2 – Minimal inhibitory concentrations of various FAS-targeted inhibitors.  *) fatty acid starvation but not killing/growth inhibition | | | | |
| --- | --- | --- | --- | --- |
|  | Target | Organism | MIC (µg/ml) | Ref |
| Cerulenin | FabF/B/H | *Escherichia coli* | 8-16 | [18, 19] |
|  |  | *Staphylococcus aureus* | 100 | [18] |
|  |  | *Staphylococcus epidermis* | 100 | [18] |
|  |  | *Staphylococcus saprophyticus* | 12.5 | [18] |
|  |  | *Sarcina lutea* | 25 | [18] |
|  |  | *Diplococcus pneumoniae* | 25 | [18] |
|  |  | *Streptococcus haemolyticus* | 50 | [18] |
|  |  | *Staphylococcus haemolyticus* | 100 | [18] |
|  |  | *Klebsiella pneumoniae* | 50 | [18] |
|  |  | *Proteus vulgaris* | 12.5 | [18] |
|  |  | *Proteus morganii* | 12.5 | [18] |
|  |  | *Salmonella typhosa* | 50 | [18] |
|  |  | *Shigella dysenteriae* | 25 | [18] |
|  |  | *Shigella flexneri* | 50 | [18] |
|  |  | *Shigella sonnei* | 50 | [18] |
|  |  | *Bacillus subtilis* | 5-12.5 | [20] |
|  |  | *Bacillus anthracis* | 62.5 | [18] |
|  |  | *Bacillus megaterium* | 50 | [18] |
|  |  | *Corynebacterium diphtheriae* | 50 | [18] |
|  |  | *Haemophilus influenzae* | 100 | [18] |
|  |  | *Mycobacterium ATCC 607* | 1.5 | [18] |
|  |  | *Mycobacterium avium* | 1.5 | [18] |
|  |  | *Mycobacterium phlei* | 3.7 | [18] |
|  |  | *Mycobacterium smegmatis* | 3.7 | [18] |
|  |  | *Mycobacterium tuberculosis* | 100 | [18] |
|  |  | *Nocardia asteroides* | 1.5 | [18] |
|  |  | *Nocardia coeliaca* | 1.5 | [18] |
|  |  | *Streptomyces griseus* | 3.1 | [18] |
|  |  | *Streptomyces lavendulae* | 1.5 | [18] |
|  |  | *Vibrio cholerae* | >200* | [21] |
|  |  | *Vibrio vulnificus* | 16.7 | [22] |
| Triclosan | FabI | *Escherichia coli* | 0.2-2 | [23] |
|  |  | *Vibrio cholerae* | 20x higher than E. coli | [7] |
|  |  | *Vibrio cholerae* |  | This work |
|  |  | *Staphylococcus aureus* | 0.025-1 | [24] |
|  |  | *Acinetobacter baumannii* | 1.5 | [25][26] |
|  |  | *Acinetobacter lwoffi* | 2 | [25] |
|  |  | *Pantoea agglomerans* | 0.13 | [25] |
|  |  | *Enterococcus clocacae* | 0.5 | [25] |
|  |  | *Klebsiella pneumoniae* | 16.5 | [25] |
|  |  | *Pseudomonas fluorescens* | 0.5 | [25] |
|  |  | *Staphylococcus aureus* | 0.06-2 | [26] |
|  |  | *Staphylococcus epidermis* | 0.09 | [26] |
|  |  | *Staphyloccus warneri* | 0.06 | [26] |
|  |  | *Enterococcus faecalis* | 10 | [23] |
|  |  | *Pseudomonas aeruginosa PA01* | 2000 | [26] |
|  |  | *Salmonella enterica* | 0.5 | [26] |
|  |  | *Mycobacterium smegmatis* | 14 | [26] |
|  |  | *Vibrio cholerae species* | 14.4 | [27] |
|  |  | *Vibrio damselae species* | 68.8 | [27] |
|  |  | *Vibrio harveyi species* | 45.3 | [27] |
|  |  | *Vibrio splendidus species* | 200 | [27] |
| Isoniazid | InhA | *Mycobacterium tuberculosis* | 0.02-0.5 | [28] |
|  |  | *Escherichia coli* | >500 | [29] |
|  |  | *Vibrio cholerae* |  | This work |
|  |  | *Salmonella typhimurium* | >500 | [29] |
|  |  | *Staphylococcus aureus* | 1.6 | [30] |
|  |  | *Enterococcus faecium* | 3.12-6.25 | [30] |

Figure S1

**
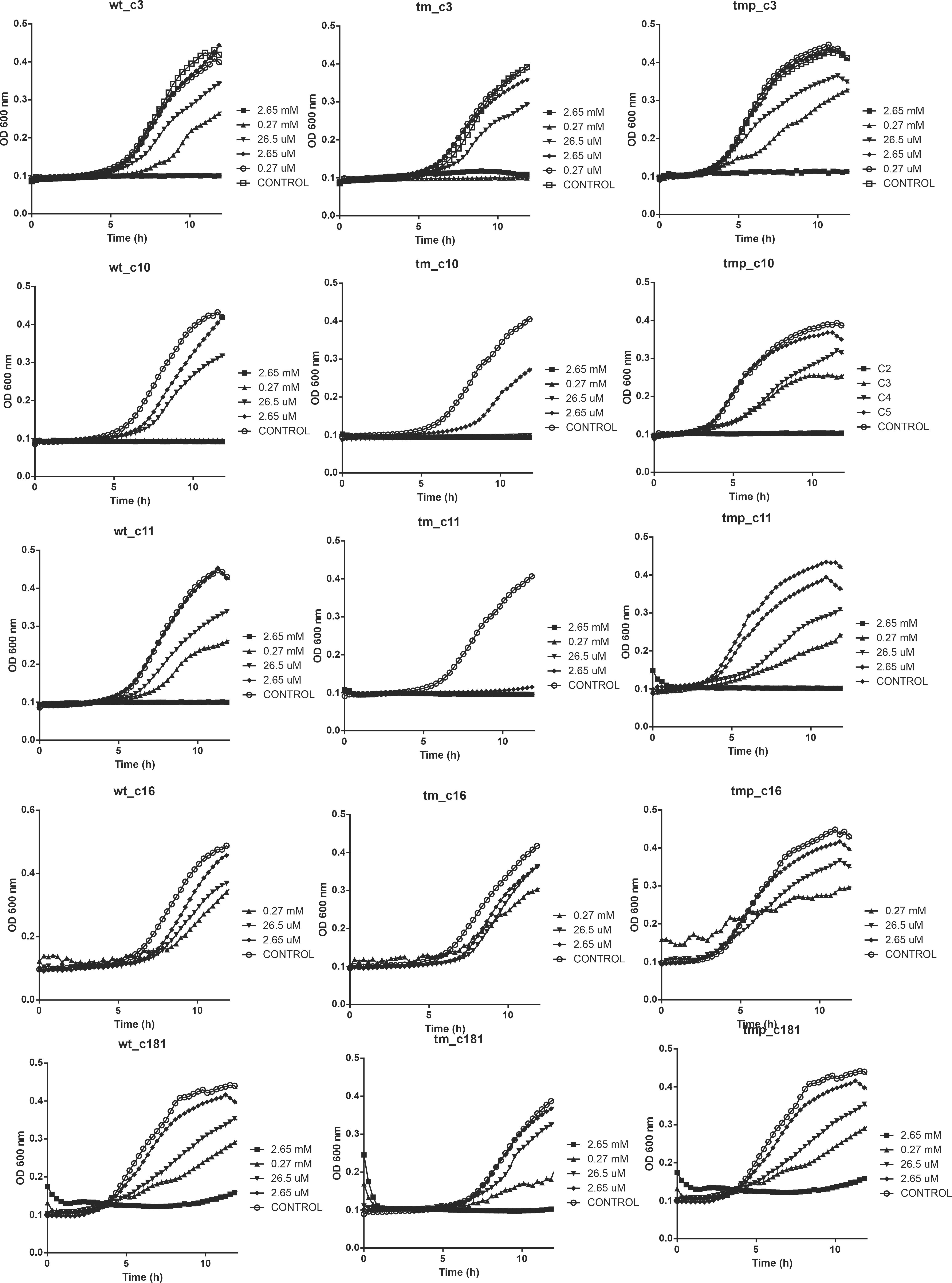
**

**Figure S1 – Representative growth curves of fatty acid supplementation to *V. cholerae* wild type, *vc2484* transposon mutant, and the same transposon mutant complemented.** Bacterial growth curves were collected on a Tecan M200Pro plate reader in 96-well plates shaking at 37 °C and absorbance measured at 600 nm. A total of 200 µl of M9 medium with 0.2 % glycerol were aliquoted per well. Overnight *V. cholerae* cultures grown in LB were inoculated at a 1:1000 dilution. Abbreviations are wt = wildtype, tm = vc2484 transposon mutant, tmp = vc2484 transposon mutant complemented with gene on plasmid, C3 = propionic acid, C10 = decanoic acid, C11 = undecanoic acid, C16 = palmitic acid, C181 = oleic acid.

Figure S2

**
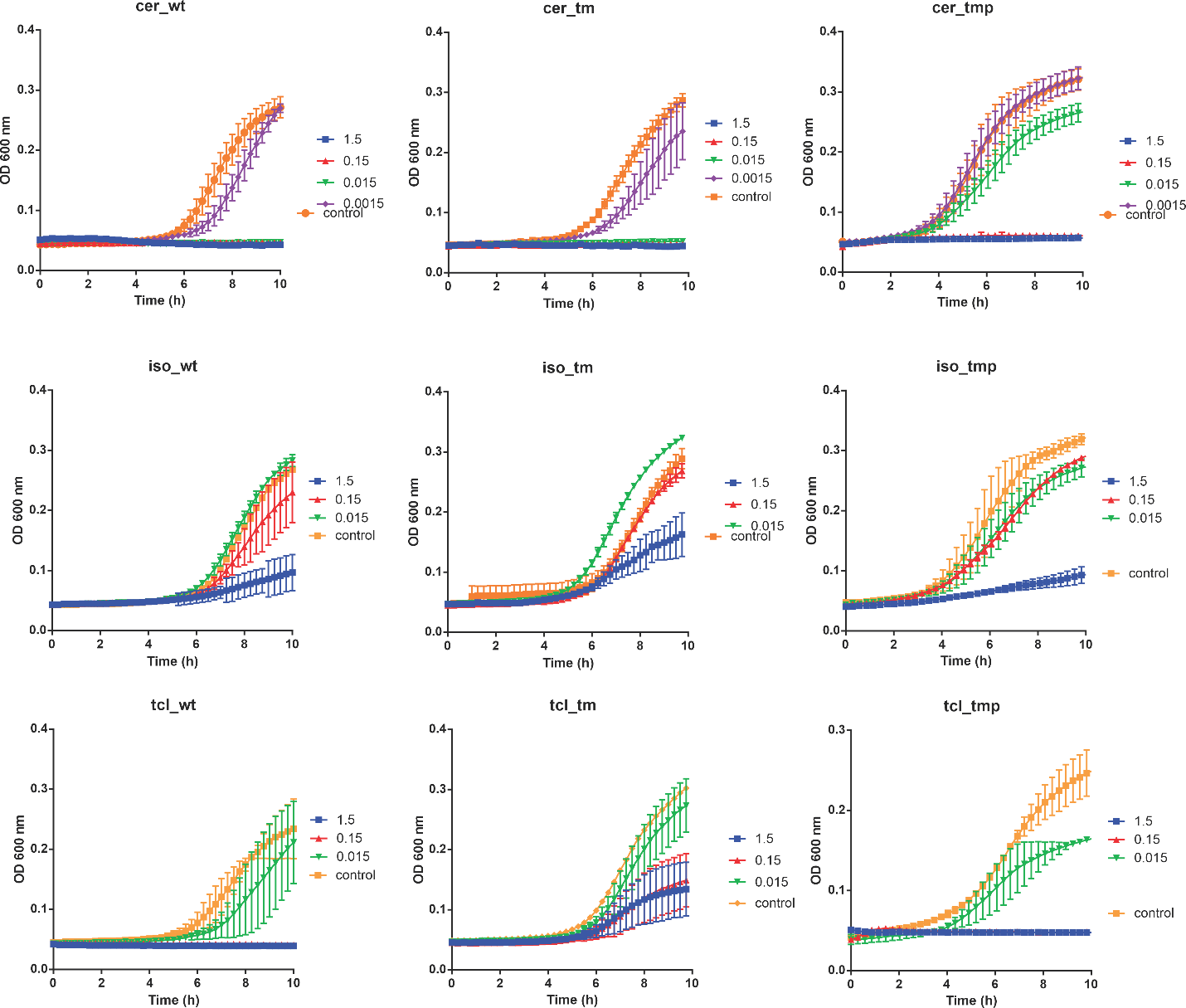
**

**Figure S2 – Growth curves of V. cholerae in the presence of FAS inhibitors.** Inhibitor concentrations in mM. Bacterial growth curves were collected on a Tecan M200Pro plate reader in 96-well plates shaking at 37 °C and absorbance measured at 600 nm. A total of 200 µl of M9 medium with 0.2 % glycerol were aliquoted per well. Overnight *V. cholerae* cultures grown in LB were inoculated at a 1:1000 dilution. Abbreviations are wt = wildtype, tm = vc2484 transposon mutant, tmp = vc2484 transposon mutant complemented with gene on plasmid, iso = isoniazid, cer = cerulenin and tcl = triclosan.

Figure S3


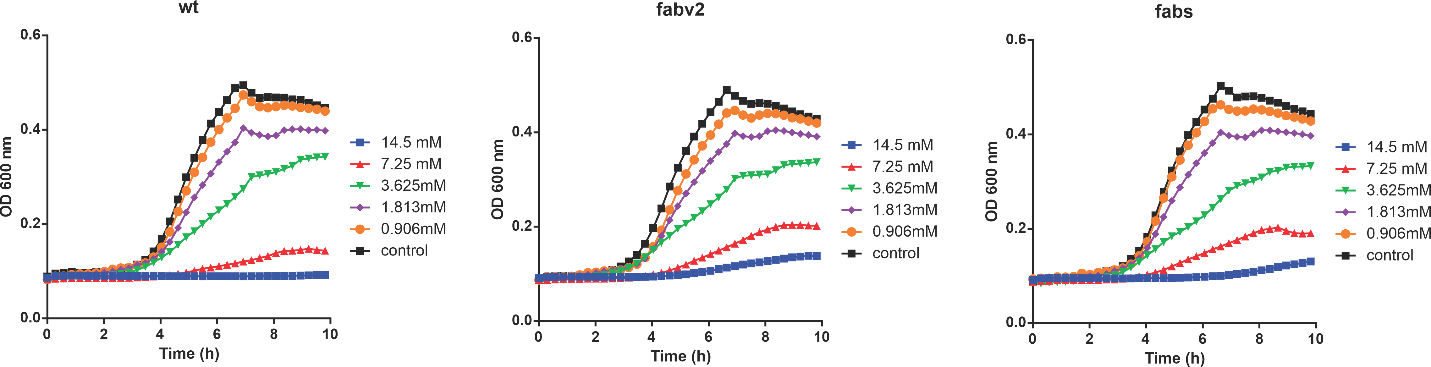


**Figure S3 – Growth curves of *V. cholerae* WT, FabV2, and FabS mutants in the presence of isoniazid.** Bacterial growth curves were collected on a Tecan M200Pro plate reader in 96-well plates shaking at 37 °C and absorbance measured at 600 nm. A total of 200 µl of M9 medium with 0.2 % glycerol were aliquoted per well. Overnight *V. cholerae* cultures grown in LB were inoculated at a 1:1000 dilution. Abbreviations are wt = wildtype, fabv2 = transposon mutant in *fabv2* and fabs = transposon mutant in *fabs*.

SI References

1. Henderson, J.C., C.M. Herrera, and M.S. Trent, *AlmG, responsible for polymyxin resistance in pandemic Vibrio cholerae, is a glycyltransferase distantly related to lipid A late acyltransferases.* Journal of Biological Chemistry, 2017. **292**(51): p. 21205-21215.

2. Kovacikova, G., W. Lin, R.K. Taylor, and K. Skorupski, *The fatty acid regulator FadR influences the expression of the virulence cascade in the El Tor biotype of Vibrio cholerae by modulating the levels of ToxT via two different mechanisms.* Journal of Bacteriology, 2017. **199**(7): p. e00762-16.

3. Merrell, D.S., D.L. Hava, and A. Camilli, *Identification of novel factors involved in colonization and acid tolerance of Vibrio cholerae.* Molecular microbiology, 2002. **43**(6): p. 1471-1491.

4. Kovacikova, G., W. Lin, and K. Skorupski, *Dual regulation of genes involved in acetoin biosynthesis and motility/biofilm formation by the virulence activator AphA and the acetate‐responsive LysR‐type regulator AlsR in Vibrio cholerae.* Molecular microbiology, 2005. **57**(2): p. 420-433.

5. Yoon, S.S. and J.J. Mekalanos, *2, 3-Butanediol synthesis and the emergence of the Vibrio cholerae El Tor biotype.* Infection and immunity, 2006. **74**(12): p. 6547-6556.

6. Lee, D., E.J. Kim, Y. Baek, J. Lee, Y. Yoon, G. Nair, S.S. Yoon, and D.W. Kim, *Alterations in glucose metabolism in Vibrio cholerae serogroup O1 El Tor biotype strains.* Scientific reports, 2020. **10**(1): p. 1-10.

7. Massengo-Tiassé, R.P. and J.E. Cronan, *Vibrio cholerae FabV defines a new class of enoyl-acyl carrier protein reductase.* Journal of Biological Chemistry, 2008. **283**(3): p. 1308-1316.

8. Brenzinger, S., L.T. van der Aart, G.P. Van Wezel, J.-M. Lacroix, T. Glatter, and A. Briegel, *Structural and proteomic changes in viable but non-culturable Vibrio cholerae.* Frontiers in Microbiology, 2019. **10**: p. 793.

9. Massengo-Tiasse, R.P., *Demonstration of novel enoyl-acyl carrier protein reductases in Vibrio cholerae*. 2011, University of Illinois at Urbana-Champaign.

10. Feng, Y. and J.E. Cronan, *Complex binding of the FabR repressor of bacterial unsaturated fatty acid biosynthesis to its cognate promoters.* Molecular microbiology, 2011. **80**(1): p. 195-218.

11. Pride, A.C., C.M. Herrera, Z. Guan, D.K. Giles, and M.S. Trent, *The outer surface lipoprotein VolA mediates utilization of exogenous lipids by Vibrio cholerae.* MBio, 2013. **4**(3).

12. Kamp, H.D., B. Patimalla-Dipali, D.W. Lazinski, F. Wallace-Gadsden, and A. Camilli, *Gene fitness landscapes of Vibrio cholerae at important stages of its life cycle.* PLoS Pathog, 2013. **9**(12): p. e1003800.

13. Mandlik, A., J. Livny, W.P. Robins, J.M. Ritchie, J.J. Mekalanos, and M.K. Waldor, *RNA-Seq-based monitoring of infection-linked changes in Vibrio cholerae gene expression.* Cell host & microbe, 2011. **10**(2): p. 165-174.

14. Yang, S., D. Xi, X. Wang, Y. Li, Y. Li, J. Yan, and B. Cao, *Vibrio cholerae VC1741 (PsrA) enhances the colonization of the pathogen in infant mice intestines in the presence of the long-chain fatty acid, oleic acid.* Microbial Pathogenesis, 2020. **147**: p. 104443.

15. Zhu, J. and J.J. Mekalanos, *Quorum sensing-dependent biofilms enhance colonization in Vibrio cholerae.* Developmental cell, 2003. **5**(4): p. 647-656.

16. Feng, Y. and J.E. Cronan, *The Vibrio cholerae fatty acid regulatory protein, FadR, represses transcription of plsB, the gene encoding the first enzyme of membrane phospholipid biosynthesis.* Molecular microbiology, 2011. **81**(4): p. 1020-1033.

17. Gao, R., J. Lin, H. Zhang, and Y. Feng, *Transcriptional repression of the VC2105 protein by Vibrio FadR suggests that it is a new auxiliary member of the fad regulon.* Applied and environmental microbiology, 2016. **82**(9): p. 2819-2832.

18. Omura, S., *The antibiotic cerulenin, a novel tool for biochemistry as an inhibitor of fatty acid synthesis.* Bacteriological reviews, 1976. **40**(3): p. 681.

19. Campbell, J.W. and J.E. Cronan, *Escherichia coli FadR positively regulates transcription of the fabB fatty acid biosynthetic gene.* Journal of bacteriology, 2001. **183**(20): p. 5982-5990.

20. Trajtenberg, F., S. Altabe, N. Larrieux, F. Ficarra, D. de Mendoza, A. Buschiazzo, and G.E. Schujman, *Structural insights into bacterial resistance to cerulenin.* The FEBS journal, 2014. **281**(10): p. 2324-2338.

21. Das, B., R.R. Pal, S. Bag, and R.K. Bhadra, *Stringent response in Vibrio cholerae: genetic analysis of spoT gene function and identification of a novel (p) ppGpp synthetase gene.* Molecular microbiology, 2009. **72**(2): p. 380-398.

22. Brown, R.N. and P.A. Gulig, *Regulation of fatty acid metabolism by FadR is essential for Vibrio vulnificus to cause infection of mice.* Journal of bacteriology, 2008. **190**(23): p. 7633-7644.

23. Zhu, L., H. Bi, J. Ma, Z. Hu, W. Zhang, J.E. Cronan, and H. Wang, *The two functional enoyl-acyl carrier protein reductases of Enterococcus faecalis do not mediate triclosan resistance.* MBio, 2013. **4**(5).

24. Peterson, K.M. and P.S. Gellings, *Multiple intraintestinal signals coordinate the regulation of Vibrio cholerae virulence determinants.* Pathogens and disease, 2018. **76**(1): p. ftx126.

25. Aiello, A.E., B. Marshall, S.B. Levy, P. Della-Latta, and E. Larson, *Relationship between triclosan and susceptibilities of bacteria isolated from hands in the community.* Antimicrobial agents and chemotherapy, 2004. **48**(8): p. 2973-2979.

26. Alfhili, M.A. and M.-H. Lee, *Triclosan: an update on biochemical and molecular mechanisms.* Oxidative medicine and cellular longevity, 2019. **2019**.

27. Lydon, K.A., M.J. Robertson, and E.K. Lipp, *Patterns of triclosan resistance in Vibrionaceae.* PeerJ, 2018. **6**: p. e5170.

28. Dickinson, J.M., V. Aber, and D. Mitchison, *Bactericidal activity of streptomycin, isoniazid, rifampin, ethambutol, and pyrazinamide alone and in combination against Mycobacterium tuberculosis.* American Review of Respiratory Disease, 1977. **116**(4): p. 627-635.

29. Rosner, J.L., *Susceptibilities of oxyR regulon mutants of Escherichia coli and Salmonella typhimurium to isoniazid.* Antimicrobial agents and chemotherapy, 1993. **37**(10): p. 2251-2253.

30. Bhikshapathi, D.V.R.N., M.S. Chandramouli, and S. Jyothisri, *Studies on different antibiotic principles against methicillin, vancomycin and linezolid resistant strains.* Int.J.Curr.Microbiol.App.Sci., 2014. **3**(12): p. 77-82.
